# Genome Sequence of Enterotoxigenic *Escherichia coli* Strain B2C

**DOI:** 10.1128/genomeA.00247-14

**Published:** 2014-04-10

**Authors:** T. P. Vipin Madhavan, Jason A. Steen, Philip Hugenholtz, Harry Sakellaris

**Affiliations:** a School of Medical Sciences, Griffith Health Centre, Griffith University, Gold Coast Campus, Queensland, Australia; b Australian Centre for Ecogenomics, School of Chemistry and Molecular Biosciences, University of Queensland, St. Lucia, Queensland, Australia

## Abstract

Enterotoxigenic *Escherichia coli* (ETEC) is a major cause of diarrheal disease around the globe, causing an estimated 380,000 deaths annually. The disease is caused by a wide variety of strains. Here, we report the genome sequence of ETEC strain B2C, which was isolated from an American soldier in Vietnam.

## GENOME ANNOUNCEMENT

Enterotoxigenic *Escherichia coli* (ETEC) causes an estimated 200 million cases of watery diarrhea and up to 380,000 deaths annually (1, 2). The major burden of disease is in developing countries lacking adequate water treatment facilities (3). The severity and symptoms of the disease can vary with the strain of ETEC causing it (4). The genomes of only two ETEC strains that infect humans, *E. coli* H10407 and E24377A, have been completely sequenced (5, 6). Strain H10407 expresses the archetypal ETEC pilus colonization factor, CFA/I, and a variety of established and putative virulence factors. E24377A expresses the colonization factors CS1 and CS3 and shares most of the same virulence genes found in H10407 (5). A draft genome of ETEC strain B7A, which expresses the CS6 colonization factor, is also available (7). B2C is an O6:H16 serotype strain of ETEC isolated in 1971 from a soldier in Vietnam (8). Since then, it has become an important laboratory strain that has been used in a variety of studies (9–13). Unlike the sequenced ETEC strains, B2C expresses CS2 and CS3 pili (9).

Genomic DNA was extracted from B2C with the Fermentas GeneJET genomic DNA purification kit (Thermo Fisher Scientific, Australia), according to the manufacturer’s protocol. One hundred nanograms of genomic DNA was used to generate a library using the Nextera DNA sample prep kit (Illumina, CA). The libraries were sequenced at the Queensland Centre for Medical Genomics, University of Queensland, Queensland (QLD), Australia, on an Illumina HiSeq 2000 sequencing system (Illumina, CA). Sequencing generated approximately 5.7 million read pairs for a ~200× coverage of the genome. The read pairs were overlapped where possible using the SeqPrep software (https://github.com/jstjohn/SeqPrep) and trimmed for quality using the Nesoni software (http://www.vicbioinformatics.com/software.nesoni.shtml). A *de novo* assembly of the overlapped and quality trimmed reads was generated using Velvet version 1.2.07 (14). The final assembly consists of 313 contigs with a total size of 5,018,127 bp. Further assembly in order to cover the entire genome was difficult due to the presence of a large number of repeat sequences and insertion elements. The size of the genome of ETEC B2C is estimated to be 5.2 Mb. The draft genome was annotated using the NCBI Prokaryotic Genome Annotation Pipeline (http://www.ncbi.nlm.nih.gov/genome/annotation_prok/) and manually curated using Artemis (15). An analysis of the genome showed that ETEC B2C has 4,990 genes, 4,804 coding sequences (CDS), 94 pseudogenes, 2 clustered regularly interspaced short palindromic repeat (CRISPR) arrays, 9 rRNAs, 83 tRNAs, and 64 frameshifted genes. Classical ETEC virulence genes, including pilus colonization factors of the chaperone usher family, and enterotoxins were also found. The genes encoding colonization factors include two operons encoding CS2 and CS3 pili and the AraC family transcriptional activator, *rns*, which control both CS2 and CS3 expression. The genome also carries e*ltA* and e*ltB*, the genes encoding the two subunits of heat-labile enterotoxin, and *sta2* and *east1*, both of which encode heat-stable enterotoxins. Since these virulence genes are carried by plasmids in other ETEC strains, we presume that they are also carried on plasmids in B2C.

### Nucleotide sequence accession number.

The draft genome sequence of enterotoxigenic *E. coli* (ETEC) 06:H16:CFA/II strain B2C was deposited in Genbank with accession no. AUZS00000000.
